# Psychological determinants of trust and reliance on automated decision-support systems: an integrated expectancy-based perspective

**DOI:** 10.3389/fpsyg.2026.1795644

**Published:** 2026-05-13

**Authors:** Usman Rehman

**Affiliations:** Graduate School of Venture, Hoseo University, Seoul, Republic of Korea

**Keywords:** affective trust, automated decision-support systems, expectancy beliefs, human–media interaction, reliance intention, technology acceptance, trust in automation

## Abstract

**Introduction:**

Automated decision-support systems are increasingly shaping human cognitive and decision-making processes, making it essential to understand the psychological mechanisms underlying users’ trust and reliance. While prior research has examined system characteristics and expectancy-based beliefs separately, limited empirical work has integrated these perspectives to explain how trust translates into reliance behavior.

**Methods:**

This study develops and tests an integrated psychological model in which affective trust mediates the relationship between cognitive evaluations and reliance intention. Drawing on trust theory, the Technology Acceptance Model, and the Unified Theory of Acceptance and Use of Technology, survey data were collected from 345 university-educated adults with prior experience using automated decision-support systems. Structural equation modeling was employed to evaluate the measurement and structural models.

**Results:**

The findings indicate that system accuracy and timeliness significantly enhance perceived reliability and responsiveness, while perceived ease of use positively influences effort expectancy and performance expectancy. Both service-related system characteristics and expectancy-based cognitive evaluations significantly contribute to affective trust formation. Affective trust evaluation, in turn, strongly predicts reliance intention, confirming its mediating role.

**Conclusion:**

The results highlight a sequential psychological process in which cognitive evaluations of system performance and usability translate into reliance through affective trust. This study contributes to the psychology of human–automation interaction by providing an integrated framework that advances theoretical understanding and offers practical guidance for designing trustworthy automated systems.

## 1 Introduction

Automated decision-support systems have become increasingly integrated into human cognitive activities, influencing how individuals process information, evaluate alternatives, and make decisions across a variety of contexts. Advances in artificial intelligence, algorithmic modeling, and interactive system design have enabled automated systems to provide real-time guidance, recommendations, and task-related support, thereby reshaping patterns of human-system interaction (Lee and See, 2004; Parasuraman et al., 2000; Hoff and Bashir, 2015). As these systems become more prevalent, understanding the psychological foundations of trust and reliance on automated systems has emerged as a critical research priority within psychology and human–media interaction scholarship (Schaefer et al., 2016; Shin, 2021; Glikson and Woolley, 2020; Rehman, 2026b).

Trust is widely recognized as a central psychological construct governing individuals’ willingness to accept and rely on automated systems. Classic theories of trust emphasize that trust reflects an individual’s willingness to become vulnerable based on positive expectations of another entity’s behavior (Mayer et al., 1995; Luhmann, 1979). When applied to technological contexts, trust is shaped not only by system performance but also by users’ subjective interpretations of system reliability, predictability, and responsiveness (McKnight et al., 2002; Gefen et al., 2003). Empirical studies consistently demonstrate that perceived reliability and responsiveness play a decisive role in fostering trust in automated environments, whereas perceived system inconsistency or opacity undermines trust and discourages reliance (Lee and See, 2004; Hoff and Bashir, 2015).

From a cognitive psychology perspective, trust in automated systems develops through expectancy-based evaluative processes. Technology acceptance research suggests that individuals form beliefs regarding system ease of use and expected performance outcomes, which subsequently influence affective evaluations and behavioral intentions (Davis, 1989; Davis et al., 1989; Venkatesh et al., 2003). These expectancy beliefs reflect users’ cognitive assessments of effort requirements and anticipated benefits, shaping whether systems are perceived as cognitively supportive or mentally burdensome (Venkatesh et al., 2012; Roca et al., 2006). In automated decision-support contexts, such cognitive evaluations determine whether users perceive systems as trustworthy partners in decision making or as tools requiring constant oversight and verification (Parasuraman et al., 2000; Schaefer et al., 2016).

Prior research has extensively examined post-adoption behavior in information systems using frameworks such as the Technology Acceptance Model (TAM; Davis, 1989), the Unified Theory of Acceptance and Use of Technology (UTAUT; Venkatesh et al., 2003), and expectation–confirmation theory (Bhattacherjee, 2001). These models collectively emphasize the importance of perceived ease of use, performance expectancy, and confirmation of expectations in shaping users’ attitudes and continued usage intentions (Thong et al., 2006; Roca et al., 2006). However, much of this literature has focused on relatively static systems, providing limited insight into how users psychologically evaluate dynamic and interactive automated systems that continuously adapt to user input and evolving task demands (Hoff and Bashir, 2015; Shin, 2021). In this context, digital transformation and system capabilities have been shown to influence organizational performance through underlying operational mechanisms (Rehman, 2026c).

Research on trust in automation further highlights that trust formation depends on both technical attributes and interactional system qualities. Meta-analytic evidence indicates that perceived reliability, transparency, and responsiveness are among the strongest predictors of trust in automated systems (Schaefer et al., 2016). Service quality research similarly emphasizes that responsiveness and timeliness are critical determinants of positive user evaluations in technology-mediated interactions (Parasuraman et al., 1988; DeLone and McLean, 2003). Despite these findings, empirical studies integrating service-related system characteristics with expectancy-based cognitive evaluations remain limited, particularly within unified psychological models of trust and reliance (Gefen et al., 2003; Hoff and Bashir, 2015).

Another notable limitation of existing research is the tendency to examine trust or reliance intention as isolated outcomes. Psychological theory suggests that trust functions as an affective mediator between cognitive system evaluations and reliance behavior. Individuals first assess system characteristics cognitively, then form affective trust judgments, and finally translate these judgments into reliance intentions (Mayer et al., 1995; McKnight et al., 2002). Expectation–confirmation theory further posits that affective evaluations emerging from prior experiences play a decisive role in shaping post-adoption behavior (Bhattacherjee, 2001; Thong et al., 2006). However, empirical studies that explicitly model this sequential psychological process in the context of automated decision-support systems remain scarce (Hoff and Bashir, 2015; Shin, 2021).

To address these gaps, the present study examines the psychological determinants of trust and reliance on automated decision-support systems by integrating system quality perceptions with expectancy-based cognitive evaluations. Drawing on established theories of trust, technology acceptance, and service quality, this study investigates how perceived system accuracy and timeliness influence reliability and responsiveness perceptions, how perceived ease of use shapes effort and performance expectancy, and how these cognitive evaluations contribute to affective trust formation. Furthermore, the study analyzes how affective trust evaluations translate into users’ intention to rely on automated systems, thereby offering a comprehensive psychological account of sustained human-system interaction.

By emphasizing core psychological mechanisms rather than application-specific outcomes, this research contributes to the Human-Media Interaction literature within *Frontiers in Psychology*. The study advances theoretical understanding of trust formation and reliance behavior in automated environments and provides empirical evidence on how cognitive and affective processes jointly shape individuals’ engagement with automated decision-support systems in increasingly algorithm-driven contexts.

## 2 Theoretical background and hypotheses development

### 2.1 Trust in automated decision-support systems

Trust is a foundational psychological construct that governs individuals’ willingness to rely on external agents under conditions of uncertainty and vulnerability (Luhmann, 1979; Mayer et al., 1995). In psychological terms, trust reflects a positive expectation regarding the intentions or performance of another entity, enabling individuals to accept risk and delegate control (Mayer et al., 1995). When applied to automated decision-support systems, trust becomes a critical determinant of whether users accept system outputs, integrate them into their cognitive processes, and rely on them for decision making (Lee and See, 2004; Hoff and Bashir, 2015).

Research in human–automation interaction consistently demonstrates that trust in automated systems is not determined solely by objective system accuracy. Instead, trust emerges from users’ subjective cognitive and affective evaluations of system behavior, including perceptions of reliability, predictability, and responsiveness (McKnight et al., 2002; Schaefer et al., 2016). When users perceive automated systems as dependable and supportive, they are more likely to develop affective trust judgments that facilitate reliance. Conversely, perceived system failures, delays, or inconsistencies undermine trust and encourage system avoidance or excessive monitoring (Lee and See, 2004; Parasuraman et al., 2000).

In this study, trust is conceptualized as an overall evaluative construct that reflects users’ psychological confidence in automated decision-support systems. While prior literature distinguishes between cognitive and affective dimensions of trust, these dimensions are closely interrelated in shaping users’ overall trust evaluations. Accordingly, this study adopts an integrated perspective in which trust represents a combined cognitive-affective evaluation formed through users’ assessments of system performance, usability, and expected outcomes. This conceptualization aligns with prior research that treats trust as a holistic psychological judgment mediating the relationship between cognitive evaluations and behavioral reliance (Mayer et al., 1995; McKnight et al., 2002).

### 2.2 System accuracy, timeliness, and service-related system characteristics

System accuracy and timeliness represent core system-level characteristics that shape users’ cognitive evaluations of automated systems. Accuracy refers to the extent to which a system provides correct and reliable outputs, while timeliness reflects the system’s ability to deliver information promptly and at appropriate moments (DeLone and McLean, 2003). Psychological research suggests that timely and accurate system responses reduce cognitive uncertainty, enhance predictability, and foster confidence in system behavior (Lee and See, 2004; Hoff and Bashir, 2015).

Service quality theory further highlights the importance of reliability and responsiveness as key interactional dimensions influencing user evaluations (Parasuraman et al., 1988). Reliability reflects the perceived consistency and dependability of system performance, whereas responsiveness captures the system’s ability to provide prompt and helpful responses to user needs. These dimensions have been shown to play a significant role in shaping trust and affective evaluations in technology-mediated environments (Gefen et al., 2003; DeLone and McLean, 2003).

In automated decision-support contexts, system accuracy and timeliness are expected to positively influence perceptions of reliability and responsiveness. When systems consistently deliver accurate outputs in a timely manner, users are more likely to perceive them as reliable and responsive, thereby fostering positive psychological evaluations. Conversely, delayed or inaccurate responses increase cognitive effort and uncertainty, undermining trust formation (Parasuraman et al., 2000; Schaefer et al., 2016).

Accordingly, the following hypotheses are proposed:

*H1:* Perceived system accuracy and timeliness positively influence perceived reliability of automated decision-support systems.

*H2:* Perceived system accuracy and timeliness positively influence perceived responsiveness of automated decision-support systems.

### 2.3 Expectancy-based cognitive evaluations

Expectancy-based cognitive evaluations play a central role in shaping individuals’ psychological responses to technology. The Technology Acceptance Model (TAM) posits that perceived ease of use significantly influences individuals’ attitudes toward technology by reducing cognitive effort and increasing perceived control (Davis, 1989; Davis et al., 1989). Systems perceived as easy to use require fewer mental resources, thereby lowering cognitive barriers to engagement and reliance.

Building on TAM, the Unified Theory of Acceptance and Use of Technology (UTAUT) introduces effort expectancy and performance expectancy as key cognitive determinants of technology acceptance (Venkatesh et al., 2003). Effort expectancy reflects the degree to which individuals believe that using a system requires minimal mental or physical effort, while performance expectancy refers to the extent to which users believe that system use will enhance task performance.

Psychological research suggests that perceived ease of use directly shapes both effort expectancy and performance expectancy by influencing users’ cognitive appraisals of system complexity and utility (Venkatesh et al., 2012; Roca et al., 2006). In automated decision-support systems, ease of use is particularly critical, as complex or unintuitive interfaces increase cognitive load and reduce perceived system value, even when technical performance is high (Parasuraman et al., 2000; Hoff and Bashir, 2015).

Thus, the following hypotheses are advanced:

*H3:* Perceived ease of use positively influences effort expectancy.

*H4:* Perceived ease of use positively influences performance expectancy.

### 2.4 Cognitive evaluations and affective trust formation

Psychological theories of trust formation emphasize that affective trust judgments are shaped by prior cognitive evaluations of competence, reliability, and expected outcomes (Mayer et al., 1995; McKnight et al., 2002). Expectancy-based theories further suggest that when individuals perceive systems as easy to use and capable of enhancing performance, they are more likely to form positive affective evaluations (Bhattacherjee, 2001; Thong et al., 2006).

In automated system contexts, effort expectancy reduces perceived cognitive costs, while performance expectancy enhances perceived instrumental value. Together, these cognitive evaluations contribute to affective trust by reinforcing beliefs that the system is supportive, dependable, and beneficial (Gefen et al., 2003; Shin, 2021). Similarly, perceptions of reliability and responsiveness strengthen trust by signaling system competence and user-oriented behavior (Lee and See, 2004; Schaefer et al., 2016).

Accordingly, the following hypotheses are proposed:

*H5:* Perceived reliability positively influences affective trust evaluation.

*H6:* Perceived responsiveness positively influences affective trust evaluation.

*H7:* Effort expectancy positively influences affective trust evaluation.

*H8:* Performance expectancy positively influences affective trust evaluation.

### 2.5 Affective trust evaluation and reliance intention

Reliance intention reflects users’ willingness to depend on automated systems for future decision making. Psychological research indicates that trust serves as a primary antecedent of reliance behavior, mediating the relationship between cognitive evaluations and behavioral outcomes (Mayer et al., 1995; Lee and See, 2004). When individuals develop affective trust in automated decision-support systems, they are more likely to rely on system outputs and integrate them into their decision-making processes.

Expectation-confirmation theory further supports this view by suggesting that affective evaluations derived from prior experiences play a decisive role in shaping post-adoption intentions (Bhattacherjee, 2001; Thong et al., 2006). In automated decision-support environments, trust functions as a psychological assurance mechanism that reduces perceived risk and uncertainty, thereby increasing reliance intention.

Thus, the following hypothesis is proposed:

*H9:* Affective trust evaluation positively influences reliance intention toward automated decision-support systems.

### 2.6 Research model

**Figure 1:**
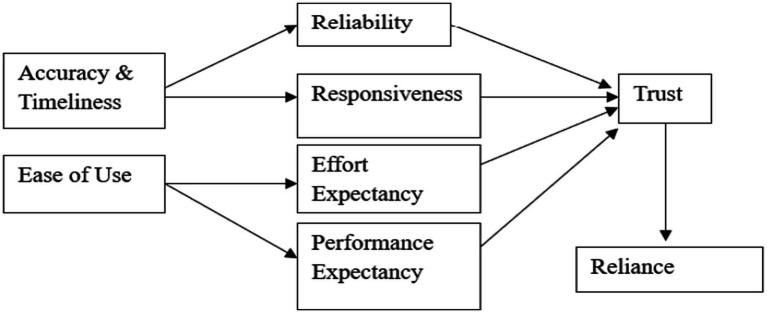
Conceptual research model.

Based on the theoretical arguments and hypotheses outlined above, Figure 1 presents the conceptual model in which system accuracy and timeliness influence perceived reliability and responsiveness, while perceived ease of use shapes effort expectancy and performance expectancy. These cognitive evaluations, in turn, contribute to affective trust formation, which subsequently predicts reliance intention. This model integrates system characteristics, expectancy-based cognitive processes, and affective trust mechanisms to provide a comprehensive psychological explanation of reliance on automated decision-support systems.

## 3 Methodology

### 3.1 Research design

This study employed a quantitative, cross-sectional survey design to examine the psychological determinants of trust and reliance on automated decision-support systems. A survey-based approach is appropriate for investigating latent psychological constructs such as trust, expectancy beliefs, and reliance intention, as it allows for the systematic measurement of cognitive and affective evaluations across a large sample (Hair et al., 2019; Nunnally and Bernstein, 1994). The proposed research model was tested using structural equation modeling (SEM), which enables simultaneous estimation of measurement and structural relationships among multiple constructs.

### 3.2 Participants and data collection

The respondents were university-educated adults with prior experience using automated decision-support systems, defined as digital systems that provide recommendations or task-related assistance for cognitive and decision-making activities. Participants were required to have regular experience with such systems to ensure meaningful evaluation of system characteristics, trust, and reliance, consistent with prior research on human-automation interaction (Lee and See, 2004; Hoff and Bashir, 2015).

Data were collected through a structured online questionnaire distributed via academic and professional networks. A total of 345 valid responses were retained after data screening. The sample included both male and female participants across a broad adult age range and diverse cultural backgrounds. The sample size was sufficient for structural equation modeling and provided adequate statistical power for testing the proposed research model (Hair et al., 2019).

### 3.3 Measures

All constructs were measured using multi-item scales adapted from well-established and validated instruments in psychology and information systems research. Items were assessed using a five-point Likert scale ranging from 1 (*strongly disagree*) to 5 (*strongly agree*). Scale adaptation followed recommended guidelines to ensure content validity and conceptual consistency with the study context (Hair et al., 2019). The full list of measurement items is provided in Appendix A.

#### 3.3.1 System accuracy and timeliness

Perceived system accuracy and timeliness were measured using items adapted from the information systems success model (DeLone and McLean, 2003). These items captured respondents’ perceptions of the system’s ability to provide correct, dependable, and timely outputs.

#### 3.3.2 Perceived ease of use

Perceived ease of use was measured using items adapted from the Technology Acceptance Model (Davis, 1989). The scale assessed the extent to which respondents perceived the system as intuitive, easy to operate, and requiring minimal mental effort.

#### 3.3.3 Reliability and responsiveness

Reliability and responsiveness were measured using items adapted from the SERVQUAL framework (Parasuraman et al., 1988). Reliability reflected perceptions of consistent and dependable system performance, while responsiveness captured perceptions of promptness and helpfulness in system responses.

#### 3.3.4 Effort expectancy and performance expectancy

Effort expectancy and performance expectancy were measured using items adapted from the Unified Theory of Acceptance and Use of Technology (UTAUT; Venkatesh et al., 2003). Effort expectancy assessed perceived ease associated with system use, whereas performance expectancy captured beliefs regarding the system’s ability to enhance task effectiveness.

#### 3.3.5 Affective trust evaluation

Affective trust evaluation was measured using items adapted from prior trust research (Mayer et al., 1995; McKnight et al., 2002). These items assessed respondents’ overall emotional confidence and positive affective evaluation toward the automated system.

#### 3.3.6 Reliance intention

Reliance intention was measured using items adapted from expectation-confirmation and post-adoption behavior literature (Bhattacherjee, 2001; Thong et al., 2006). The scale captured respondents’ intention to depend on automated decision-support systems in future interactions.

### 3.4 Data analysis procedure

Data screening and preliminary analyses were conducted using SPSS. The measurement and structural models were subsequently tested using structural equation modeling (SEM) with AMOS. Prior to hypothesis testing, data were screened for missing values, normality, and outliers following established guidelines (Hair et al., 2019).

The measurement model was evaluated by examining internal consistency reliability, convergent validity, and discriminant validity. Reliability was assessed using Cronbach’s alpha and composite reliability values, while convergent validity was evaluated through average variance extracted (AVE). Discriminant validity was assessed using both the Fornell–Larcker criterion (Fornell and Larcker, 1981) and the Heterotrait-Monotrait ratio (HTMT), providing a more rigorous assessment of construct distinctiveness.

Subsequently, the structural model was tested to examine the hypothesized relationships among constructs. Model fit was assessed using multiple goodness-of-fit indices, including the comparative fit index (CFI), Tucker–Lewis index (TLI), root mean square error of approximation (RMSEA), and standardized root mean square residual (SRMR), in accordance with recommended thresholds (Bentler, 1990; Browne and Cudeck, 1993).

## 4 Results

### 4.1 Sample characteristics

The demographic characteristics of the respondents are summarized in Table 1. The final sample consisted of 345 participants, of whom 57.4% were male (*n* = 198) and 42.6% were female (*n* = 147). Regarding age, 27.8% of respondents were aged 18–25, 41.2% were 26–35, 20.6% were 36–45, and 10.4% were above 45 years old.

**Table 1:** Demographic characteristics of respondents (*N* = 345).

Characteristic	Category	Frequency	Percentage (%)
Gender	Male	198	57.4
	Female	147	42.6
Age	18–25	96	27.8
	26–35	142	41.2
	36–45	71	20.6
	Above 45	36	10.4
Experience with automated systems	Moderate	201	58.3
	Extensive	144	41.7

In terms of experience with automated decision-support systems, 58.3% of participants reported moderate experience, while 41.7% indicated extensive experience. Overall, the sample represents a diverse group of adult users with sufficient familiarity with automated decision-support systems, supporting the validity of subsequent analyses.

### 4.2 Measurement model assessment

Prior to testing the hypothesized structural relationships, the measurement model was evaluated to assess internal consistency reliability, convergent validity and discriminant validity.

#### 4.2.1 Internal consistency reliability and convergent validity

Internal consistency reliability was examined using Cronbach’s alpha (*α*) and composite reliability (CR), while convergent validity was assessed using average variance extracted (AVE). As shown in Table 2, Cronbach’s alpha values ranged from 0.84 to 0.91, exceeding the recommended threshold of 0.70. Composite reliability values ranged from 0.88 to 0.93, further confirming satisfactory internal consistency. Convergent validity was also established, as all AVE values exceeded the recommended minimum of 0.50, ranging from 0.65 to 0.77. These results indicate that each construct explained a substantial proportion of variance in its measurement items, supporting adequate convergent validity. All standardized factor loadings exceeded the recommended threshold of 0.60, indicating adequate indicator reliability. Detailed item-level standardized loadings are provided in Appendix B.

**Table 2:** Measurement model: reliability and convergent validity.

Construct	Items	Cronbach’s *α*	CR	AVE
System accuracy & timeliness	4	0.87	0.90	0.69
Perceived ease of use	4	0.88	0.91	0.72
Reliability	4	0.86	0.89	0.67
Responsiveness	4	0.85	0.88	0.65
Effort expectancy	3	0.84	0.88	0.71
Performance expectancy	4	0.89	0.92	0.74
Affective trust evaluation	4	0.91	0.93	0.77
Reliance intention	3	0.88	0.91	0.75

#### 4.2.2 Discriminant validity

Discriminant validity was assessed using the Fornell–Larcker criterion. As presented in Table 3, the square root of the AVE for each construct (diagonal values) was greater than its correlations with all other constructs. This finding indicates that the constructs are empirically distinct and measure unique psychological concepts, thereby confirming discriminant validity. In addition, discriminant validity was further assessed using the Heterotrait–Monotrait ratio (HTMT).

**Table 3:** Discriminant validity (Fornell–Larcker criterion).

Construct	SAT	PEOU	REL	RES	EE	PE	TRUST	RI
SAT	**0.83**							
PEOU	0.46	**0.85**						
REL	0.52	0.48	**0.82**					
RES	0.49	0.45	0.57	**0.81**				
EE	0.41	0.54	0.39	0.42	**0.84**			
PE	0.44	0.59	0.43	0.45	0.61	**0.86**		
TRUST	0.55	0.51	0.63	0.60	0.48	0.66	**0.88**	
**RI**	0.49	0.46	0.57	0.54	0.44	0.58	0.71	**0.87**

Diagonal elements (bold values) represent the square root of the Average Variance Extracted (AVE) for each construct. Off-diagonal elements represent the correlations between constructs. Discriminant validity is established when the square root of AVE for each construct exceeds its correlations with other constructs (Fornell & Larcker, 1981).

As shown in Table 4, all HTMT values are below the recommended threshold of 0.85, indicating satisfactory discriminant validity. These results confirm that the constructs are empirically distinct despite moderate correlations among related variables.

**Table 4:** Heterotrait–Monotrait Ratio (HTMT).

Construct	SAT	PEOU	REL	RES	EE	PE	TRUST	RI
SAT	—							
PEOU	0.72	—						
REL	0.68	0.70	—					
RES	0.66	0.69	0.75	—				
EE	0.65	0.73	0.64	0.66	—			
PE	0.67	0.76	0.69	0.70	0.78	—		
TRUST	0.70	0.72	0.80	0.78	0.68	0.82	—	
RI	0.65	0.68	0.74	0.72	0.66	0.76	0.84	—

#### 4.2.3 Common method bias

Given the self-reported nature of the data, potential common method bias was assessed using both Harman’s single-factor test and the common latent factor (CLF) approach, as recommended by Podsakoff et al. (2003).

First, Harman’s single-factor test was conducted using unrotated exploratory factor analysis. The results revealed that multiple factors emerged, and the first factor accounted for 32.4% of the total variance, which is below the recommended threshold of 50%. This indicates that no single factor dominates the variance, suggesting that common method bias is not a serious concern (Podsakoff et al., 2003).

Second, a common latent factor (CLF) was included in the measurement model to capture the potential shared variance among all observed indicators. All measurement items were allowed to load onto both their respective theoretical constructs and the latent method factor. The comparison between models with and without the CLF showed that the differences in standardized factor loadings were minimal (all differences < 0.05), which is below the commonly accepted threshold, indicating that common method variance does not significantly influence the parameter estimates (Podsakoff et al., 2003).

Taken together, the results from both diagnostic techniques provide converging evidence that common method bias is unlikely to threaten the validity and robustness of the study’s findings.

### 4.3 Structural model and hypothesis testing

After confirming the adequacy of the measurement model, the structural model was evaluated to test the proposed hypotheses. Structural equation modeling was employed to estimate the relationships among constructs. Model fit was assessed using multiple goodness-of-fit indices. The results indicated an acceptable model fit (CFI = 0.94, TLI = 0.93, RMSEA = 0.045, SRMR = 0.041), consistent with recommended thresholds (Bentler, 1990; Browne and Cudeck, 1993).

The results of hypothesis testing are summarized in Table 5. System accuracy and timeliness had a significant positive effect on perceived reliability (*β* = 0.53, *t* = 9.84, *p* < 0.001), supporting H1, and on perceived responsiveness (*β* = 0.49, *t* = 8.91, *p* < 0.001), supporting H2. These findings indicate that accurate and timely system performance plays a critical role in shaping users’ perceptions of key service-related system characteristics.

Consistent with technology acceptance theory, perceived ease of use exerted a strong positive influence on effort expectancy (*β* = 0.61, *t* = 11.22, *p* < 0.001), supporting H3, and on performance expectancy (*β* = 0.64, *t* = 12.07, *p* < 0.001), supporting H4. This suggests that systems perceived as intuitive and easy to use reduce perceived effort while enhancing expectations of performance benefits.

**Table 5:** Structural model results and hypothesis testing.

Hypothesis	Path	*β*	*t*-value	*p*-value	Result
H1	Accuracy & timeliness → reliability	0.53	9.84	<0.001	Supported
H2	Accuracy & timeliness → responsiveness	0.49	8.91	<0.001	Supported
H3	Ease of use → effort expectancy	0.61	11.22	<0.001	Supported
H4	Ease of use → performance expectancy	0.64	12.07	<0.001	Supported
H5	Reliability → trust	0.32	6.18	<0.001	Supported
H6	Responsiveness → trust	0.28	5.64	<0.001	Supported
H7	Effort Expectancy → trust	0.19	3.92	<0.01	Supported
H8	Performance expectancy → trust	0.41	7.88	<0.001	Supported
H9	Trust → reliance intention	0.71	14.56	<0.001	Supported

The analysis further revealed that perceived reliability significantly enhanced affective trust evaluation (*β* = 0.32, *t* = 6.18, *p* < 0.001), supporting H5, and perceived responsiveness also positively influenced affective trust evaluation (*β* = 0.28, *t* = 5.64, *p* < 0.001), supporting H6. These results underscore the importance of dependable and responsive system behavior in fostering users’ emotional confidence in automated decision-support systems.

Expectancy-based cognitive evaluations also contributed significantly to trust formation. Effort expectancy had a positive effect on affective trust evaluation (*β* = 0.19, *t* = 3.92, *p* < 0.01), supporting H7, while performance expectancy exhibited a strong positive relationship with affective trust evaluation (*β* = 0.41, *t* = 7.88, *p* < 0.001), supporting H8.

Finally, affective trust evaluation emerged as a strong predictor of reliance intention (*β* = 0.71, *t* = 14.56, *p* < 0.001), supporting H9. This result highlights the central role of trust as a psychological mechanism through which cognitive evaluations and system perceptions translate into users’ intention to rely on automated decision-support systems.

Overall, the structural model results provide robust empirical support for the proposed research framework, demonstrating that system characteristics and expectancy-based cognitive evaluations jointly influence affective trust, which in turn strongly predicts reliance intention.

In addition to the direct structural relationships, the mediating role of affective trust was further examined. As shown in Table 6. To provide a comprehensive assessment of the mediating role of affective trust, both direct, indirect, and total effects were examined using a bootstrapping approach with 5,000 resamples and bias-corrected confidence intervals. In addition to the indirect effects, direct paths from reliability, responsiveness, effort expectancy, and performance expectancy to reliance intention were included to determine the nature of mediation.

**Table 6:** Mediation analysis: direct, indirect, total effects, and VAF.

Path	Direct effect (*β*)	Indirect effect (*β*)	Total effect (*β*)	VAF (%)	Mediation type
Reliability → trust → reliance	0.22	0.18	0.40	45%	Partial
Responsiveness → trust → reliance	0.20	0.16	0.36	44%	Partial
Effort Expectancy → trust → reliance	0.19	0.14	0.33	42%	Partial
Performance expectancy → trust → reliance	0.25	0.21	0.46	46%	Partial

The results indicate that all indirect effects through affective trust are statistically significant, as their confidence intervals do not include zero. The direct effects were also statistically significant (*p* < 0.05), indicating that the independent variables exert both direct and indirect influences on reliance intention. To assess the magnitude of mediation, Variance Accounted For (VAF) was calculated. The VAF values ranged from 42 to 46%, indicating partial mediation across all examined relationships. These findings suggest that affective trust serves as a key psychological mechanism through which cognitive evaluations influence reliance intention, while the direct effects indicate that these factors also independently contribute to reliance behavior.

This pattern of partial mediation aligns with prior trust and technology acceptance literature, which suggests that cognitive evaluations can influence behavioral intentions both directly and indirectly through affective trust mechanisms.

## 5 Discussion

The present study investigated the psychological mechanisms underlying trust and reliance on automated decision-support systems by integrating system characteristics with expectancy-based cognitive evaluations. Drawing on trust theory, technology acceptance research, and human–media interaction literature, the study proposed and empirically tested a comprehensive model explaining how users’ cognitive assessments shape affective trust and, in turn, reliance intention. Overall, the findings provide strong support for the proposed framework and offer several important theoretical and practical insights, contributing to the growing body of research on AI-enabled decision-making and human–system interaction (Rehman, 2026b).

First, the results demonstrate that system accuracy and timeliness play a critical role in shaping users’ perceptions of reliability and responsiveness. These findings are consistent with prior research suggesting that dependable and timely system performance reduces uncertainty and enhances predictability, which are essential conditions for trust formation in automated environments (Lee and See, 2004; Hoff and Bashir, 2015). By empirically confirming these relationships, the study reinforces the view that trust in automated systems is grounded not only in technical performance but also in users’ psychological interpretations of system behavior.

Second, the findings highlight the importance of perceived ease of use as a key antecedent of effort expectancy and performance expectancy. Systems perceived as intuitive and easy to operate were associated with lower perceived cognitive effort and higher expectations of performance benefits. This result aligns with the Technology Acceptance Model and UTAUT, which emphasize the central role of ease of use in shaping expectancy beliefs (Davis, 1989; Venkatesh et al., 2003). In the context of automated decision-support systems, ease of use appears particularly important because complex interfaces may increase cognitive load and undermine positive evaluations, even when system outputs are accurate.

Third, the study provides robust evidence that affective trust evaluation is jointly shaped by both service-related system characteristics (reliability and responsiveness) and expectancy-based cognitive evaluations (effort and performance expectancy). The relatively modest effect of effort expectancy suggests that usability plays a secondary role compared to performance-related evaluations in shaping trust. Among these predictors, performance expectancy and reliability exhibited relatively stronger effects on trust, suggesting that users’ emotional confidence in automated systems is closely tied to perceived competence and anticipated utility. This finding supports psychological theories that conceptualize trust as an affective response emerging from prior cognitive assessments (Mayer et al., 1995; McKnight et al., 2002).

Most importantly, the results provide empirical support for the mediating role of affective trust in translating cognitive evaluations into reliance intention. Affective trust evaluation emerged as a strong predictor of users’ intention to rely on automated decision-support systems, underscoring trust’s function as a psychological assurance mechanism that reduces perceived risk and facilitates reliance behavior. This finding is consistent with prior research on trust in automation and human–AI interaction (Schaefer et al., 2016; Shin, 2021; Miller, 2019) and extends it by empirically demonstrating a sequential process in which system characteristics and expectancy beliefs shape reliance through affective trust. These findings are also consistent with prior research on AI-enabled decision-making and system-supported operational effectiveness (Rehman, 2026a; Rehman, 2026b). The mediation analysis further revealed that affective trust exhibits partial mediation across all relationships, indicating that while trust serves as a central psychological mechanism, cognitive evaluations also retain a direct and significant influence on reliance intention.

### 5.1 Theoretical implications

This study makes several important contributions to the human-media interaction and psychology of automation literature. First, it advances trust research by integrating service-related system characteristics with expectancy-based cognitive evaluations in a single psychological model. While prior studies have often examined these factors separately, the present research demonstrates how they jointly contribute to affective trust formation.

Second, the study extends technology acceptance theories by emphasizing the affective dimension of trust as a key psychological mechanism linking cognition to behavior. Rather than treating reliance intention as a direct outcome of cognitive beliefs, the findings highlight the importance of affective trust as an intermediary process, thereby offering a more nuanced explanation of post-adoption behavior in automated systems.

Third, by focusing on automated decision-support systems in general rather than specific applications, the study contributes to a broader psychological understanding of trust and reliance in increasingly algorithm-driven environments. This generalizable perspective enhances the theoretical relevance of the findings across different domains of human-system interaction.

### 5.2 Practical implications

The findings also offer several practical implications for designers and developers of automated decision-support systems. First, enhancing system accuracy and response timeliness is essential not only for technical performance but also for fostering positive psychological evaluations of reliability and responsiveness. Second, designers should prioritize usability and interface simplicity, as perceived ease of use plays a critical role in shaping expectancy beliefs and subsequent trust.

Additionally, system features that clearly communicate performance benefits and support users’ tasks can strengthen performance expectancy, thereby enhancing affective trust and reliance. By addressing both cognitive and affective dimensions of user experience, practitioners can design automated systems that are more likely to be trusted and relied upon over time.

### 5.3 Limitations and future research

Despite its contributions, this study has several limitations that suggest directions for future research. First, the cross-sectional research design limits causal inference. Future studies could employ longitudinal or experimental designs to examine how trust and reliance evolve over time. Second, data were collected using self-reported measures, which may be subject to response bias. Future research could incorporate behavioral or objective usage data to complement subjective evaluations. The sample composition, consisting largely of relatively experienced and younger users, may have influenced the observed relationships, particularly by emphasizing performance-related evaluations in trust formation.

Finally, although the study focused on general automated decision-support systems, future research could explore boundary conditions by examining how individual differences, task complexity, or contextual factors influence trust formation and reliance behavior. Such extensions would further enrich the psychological understanding of human–automation interaction.

Another limitation relates to the simplified structure of the reliance intention model. In the present study, reliance intention is primarily explained through affective trust, which demonstrated a strong effect. However, prior research on human–automation interaction suggests that reliance behavior may also be influenced by additional factors such as perceived risk, task complexity, and accountability considerations. Future research is encouraged to extend the model by incorporating these factors to provide a more comprehensive understanding of reliance behavior in automated decision-support contexts.

## 6 Conclusion

This study examined the psychological determinants of trust and reliance on automated decision-support systems by integrating system characteristics with expectancy-based cognitive evaluations. Drawing on established theories of trust, technology acceptance, and human-media interaction, the study proposed and empirically tested a comprehensive psychological model explaining how users’ cognitive assessments shape affective trust and, in turn, reliance intention. The findings provide clear evidence that trust serves as a key psychological mechanism through which perceptions of system performance and usability translate into reliance on automated systems.

The results highlight the importance of system accuracy, timeliness, reliability, and responsiveness in shaping users’ psychological evaluations of automated decision-support systems. In addition, expectancy-based cognitive beliefs-particularly perceived ease of use and performance expectancy were shown to play a critical role in fostering affective trust. By demonstrating that affective trust strongly predicts reliance intention, the study underscores the need to consider both cognitive and emotional processes when examining human–automation interaction.

From a theoretical perspective, this research contributes to the psychology literature by offering an integrated framework that connects system characteristics, cognitive expectancy beliefs, affective trust, and reliance behavior. The findings extend existing models of technology acceptance and trust by explicitly modeling trust as an affective mediator, thereby providing a more nuanced understanding of post-adoption behavior in automated environments.

Practically, the study suggests that developers and designers of automated decision-support systems should focus not only on technical performance but also on usability and interaction quality to foster trust and reliance. Systems that are accurate, responsive, and easy to use are more likely to be perceived as cognitively supportive and emotionally trustworthy, increasing the likelihood of sustained reliance.

In conclusion, as automated decision-support systems continue to shape human decision-making processes, understanding the psychological foundations of trust and reliance becomes increasingly important. By elucidating these mechanisms, the present study offers valuable insights for both theory and practice and provides a foundation for future research on human-system interaction in algorithm-driven contexts.

## Data Availability

The raw data supporting the conclusions of this article will be made available by the authors, without undue reservation.
