# Supplementary material for: Psychological determinants of trust and reliance on automated decision-support systems: an integrated expectancy-based perspective
Source: Front Psychol. 2026 May 13;17:1795644. doi: 10.3389/fpsyg.2026.1795644 (PMC13212112; doi:10.3389/fpsyg.2026.1795644)
Supplement: Supplementary file 1 [file Supplementary_file_1.docx]

**Appendix A. Measurement Items**

All items were measured using a five-point Likert scale (1 = strongly disagree, 5 = strongly agree).

System Accuracy and Timeliness (adapted from DeLone & McLean, 2003)

- The system provides accurate results.
- The system delivers information in a timely manner.
- The outputs generated by the system are reliable.
- The system responds quickly to user requests.

Perceived Ease of Use (adapted from Davis, 1989)

- The system is easy to use.
- Learning to operate the system is straightforward for me.
- The system is clear and understandable.
- It is easy for me to become skillful in using the system.

Reliability (adapted from Parasuraman et al., 1988)

- The system performs consistently.
- The system is dependable in its operation.
- The system functions as expected.
- The system provides reliable service.

Responsiveness (adapted from Parasuraman et al., 1988)

- The system responds promptly to my inputs.
- The system provides timely assistance when needed.
- The system is quick to address my requests.
- The system is helpful when I require support.

Effort Expectancy (adapted from Venkatesh et al., 2003)

- Using the system requires minimal effort.
- It is easy for me to use the system.
- I find the system simple to operate.

Performance Expectancy (adapted from Venkatesh et al., 2003)

- Using the system improves my performance.
- The system enhances my effectiveness in completing tasks.
- The system helps me accomplish tasks more quickly.
- The system increases my productivity.

Affective Trust Evaluation (adapted from Mayer et al., 1995; McKnight et al., 2002)

- I trust the system.
- I feel confident in the system.
- The system is trustworthy.
- I feel secure when relying on the system.

Reliance Intention (adapted from Bhattacherjee, 2001; Thong et al., 2006)

- I intend to rely on the system in the future.
- I plan to use the system for decision-making tasks.
- I depend on the system when performing my tasks.

**Appendix B. Confirmatory Factor Analysis (CFA) Item Loadings**

The table below reports the standardized factor loadings obtained from the confirmatory factor analysis (CFA) for all measurement items.

| **Construct** | **Item** | **Standardized Loading** |
| --- | --- | --- |
| System Accuracy and Timeliness | SAT1 | 0.82 |
|  | SAT2 | 0.85 |
|  | SAT3 | 0.80 |
|  | SAT4 | 0.83 |
| Perceived Ease of Use | PEOU1 | 0.86 |
|  | PEOU2 | 0.88 |
|  | PEOU3 | 0.84 |
|  | PEOU4 | 0.87 |
| Reliability | REL1 | 0.81 |
|  | REL2 | 0.84 |
|  | REL3 | 0.79 |
|  | REL4 | 0.83 |
| Responsiveness | RES1 | 0.80 |
|  | RES2 | 0.82 |
|  | RES3 | 0.78 |
|  | RES4 | 0.81 |
| Effort Expectancy | EE1 | 0.83 |
|  | EE2 | 0.86 |
|  | EE3 | 0.84 |
| Performance Expectancy | PE1 | 0.87 |
|  | PE2 | 0.89 |
|  | PE3 | 0.85 |
|  | PE4 | 0.88 |
| Affective Trust Evaluation | TRUST1 | 0.88 |
|  | TRUST2 | 0.90 |
|  | TRUST3 | 0.87 |
|  | TRUST4 | 0.91 |
| Reliance Intention | RI1 | 0.86 |
|  | RI2 | 0.88 |
|  | RI3 | 0.85 |
